# Correction: Occupational and geographical differentials in financial protection against healthcare out-of-pocket payments in Nepal: Evidence for universal health coverage

**DOI:** 10.1371/journal.pone.0334765

**Published:** 2025-10-20

**Authors:** The PLOS One Editors

## Notice of Republication

This article [1] was republished on October 2, 2025, to address an issue identified post-publication and to make the following amendments:

- The article’s Data Availability statement is updated to: All data used in this study are publicly available and can be obtained upon request from the National Statistics office of the Government of Nepal (https://microdata.nsonepal.gov.np/index.php/home).
- The 5^th^ sentence of the ‘Data Source’ subsection of the Materials and method section is updated to: We used information from Sections 1, 3, 4, and 5 of the questionnaires in [40] that covered socio-demographic variables, the food and non-food consumption and healthcare OOP payments, and information on economic activities and occupations, respectively.

Please download this article again to view the correct version.
