# Supplementary material for: Occupational and geographical differentials in financial protection against healthcare out-of-pocket payments in Nepal: Evidence for universal health coverage
Source: PLoS One. 2023 Jan 27;18(1):e0280840. doi: 10.1371/journal.pone.0280840 (PMC9882914; doi:10.1371/journal.pone.0280840)
Supplement: S1 File — (DOCX) [file pone.0280840.s001.docx]

We used following supporting information/resources and files; they are available upon request from the concerned authority. Find the url for the resources.

- AHS 2014_15_Manual for enumerator & supervisor_Nepali
- AHS2014_15_Questionaire_English (1)
- Annual Household Survey Report 2014-15

<https://microdata.cbs.gov.np/index.php/catalog/66>

<https://cbs.gov.np/publications/>
