# Supplementary material for: Occupational and geographical differentials in financial protection against healthcare out-of-pocket payments in Nepal: Evidence for universal health coverage
Source: PLoS One. 2023 Jan 27;18(1):e0280840. doi: 10.1371/journal.pone.0280840 (PMC9882914; doi:10.1371/journal.pone.0280840)
Supplement: Attachment — Submitted filename: Comments.docx [file pone.0280840.s002.docx]

The study investigates financial protection using two approaches in Nepal. They disaggregate their analysis based on occupations and geography.

1. Cross-sectional AHS 2014-15: Authors use one-year cross-section data to do their analysis. Using one-year data would not tell us much about what is going on with respect to financial protection. If possible, I would suggest adding more past data to see the country's CHE trend over the years.
2. The authors suggest designing risk-pooling arrangements to make progress towards UHC. Will this solve all the problems people face in Nepal, especially the most disadvantaged groups incurring CHE? How about the supply side of the health care system? If there are not enough provisions for these services, how come, UHC will help solve CHE? I would recommend talking about the supply side of the health care system in Nepal.
3. Introduction: "In the last few decades, many low-and middle-income countries (LMIC) have been reforming their health financing arrangements for UHC (4, 5). Countries such as Thailand, Ghana, Tanzania, and Indonesia have successfully introduced the risk-pooling arrangements to the occupation groups of both the formal and informal sector of economy (4-6)."
   a. Turkey introduced universal health coverage in 2008 and offered a good example of such countries that implemented UHC successfully. Therefore, I would suggest including the below references in the text as well to mention Turkey's experience:

Ti̇rgi̇l, A., & Özbuğday, F. C. (2020). Does Public Health Insurance Provide Financial Protection Against Out-Of-Pocket Health Payments? Evidence from Turkey. *Sosyoekonomi*, *28*(45), 11-24.

Atun, R., Aydın, S., Chakraborty, S., Sümer, S., Aran, M., Gürol, I., ... & Akdağ, R. (2013). Universal health coverage in Turkey: enhancement of equity. *The Lancet*, *382*(9886), 65-99.

4. "There are two commonly used approaches to measure the extent of financial hardship and financial protection in healthcare: (i) catastrophic health expenditure (CHE) and (ii)impoverishment due to healthcare out-of-pocket (OOP) payments."

Other studies in the literature look at causal mechanisms in terms of catastrophic health expenditures. Therefore, I suggest that the authors provide references to the relevant literature. For example,

Miller, G., Pinto, D. M., & Vera-Hernández, M. (2009). *Risk protection, service use, and health outcomes under Colombia's health insurance program for the poor* (No. w15456). National Bureau of Economic Research.

5. There is much research concerning CHE using these two approaches authors utilize. I do not see any contribution to the literature regarding the approaches taken. However, the authors state that it is the first study to look at CHE, disaggregated at occupations and regions in South-East Asia, which can be accepted as a contribution.
