# Supplementary material for: Occupational and geographical differentials in financial protection against healthcare out-of-pocket payments in Nepal: Evidence for universal health coverage
Source: PLoS One. 2023 Jan 27;18(1):e0280840. doi: 10.1371/journal.pone.0280840 (PMC9882914; doi:10.1371/journal.pone.0280840)
Supplement: Attachment — Submitted filename: Letter cum response to reviewers.docx [file pone.0280840.s003.docx]

Date: 02/10/2022

To,

The Academic Editor

PLOS One

**Subject: Rebuttal letter to the manuscript PONE-D-22-17102.**

Dear Sir

Thank you very much for a thorough review by the academic editor and reviewers – the points raised provided us an opportunity to improve the manuscript. Below, we have provided a point-by-point response to the issues raised by the academic editor and reviewers. The comments are in normal font, our response is in blue font and in-text additions to address the comments are in blue italic fonts.

**Response to Editor’s remarks**

1. Please ensure that your manuscript meets PLOS ONE's style requirements, including those for file naming.

**Response:** We have now changed the manuscript formatting that meets PLOS ONE style.

2. In your Data Availability statement, you have not specified where the minimal data set underlying the results described in your manuscript can be found.

**Response:** We have attached the raw data as supporting information file-I, that covers data, survey report, questionnaire and field manual.

3. Please include your full ethics statement in the ‘Methods’ section of your manuscript file. In your statement, please include the full name of the IRB or ethics committee who approved or waived your study, as well as whether or not you obtained informed written or verbal consent. If consent was waived for your study, please include this information in your statement as well.

**Response:** This paper is based on a social survey conducted by Central Bureau of Statistics, Nepal, and it is currently publicly available for further analysis. We have now included ethical considerations in the method section as follows.

…. This study is based on a publicly available dataset. The permission to access and use this dataset was obtained from Central Bureau of Statistics (CBS), Government of Nepal. The protocol for the survey was approved by the Central Bureau of Statistics (CBS) based on the Statistical Act (1958) in September 2018. This Act enables CBS to implement surveys as per the Government of Nepal’s ethics protocol without involving an institutional review board (IRB) approval. So, all procedures were performed in accordance with relevant guidelines. During the data collection, verbal consent was obtained from each respondent after a thorough introduction of the survey. All respondents were briefed about the voluntary nature of participation. Participants were assured anonymity and confidentiality of shared information.

Besides this, considering the editor’s emphasis on the second reviewer’s remark – prepare a cohesive story from the result section – we given attention to this point and revised the respective sections accordingly.

**RESPONSE TO THE COMMENTS FROM REVIEWER 1**

The study investigates financial protection using two approaches in Nepal. They disaggregate their analysis based on occupations and geography.

1.	Cross-sectional AHS 2014-15: Authors use one-year cross-section data to do their analysis. Using one-year data would not tell us much about what is going on with respect to financial protection. If possible, I would suggest adding more past data to see the country's CHE trend over the years.

**Response:** Many thanks for this important suggestion. Our aim was to examine the financial protection indicators, both levels and distribution for the most recent annual household survey (AHS) that measures both consumption and occupational details, thus disaggregation at occupation and geography was possible. There are additional reasons why this round of AHS was used. In terms of timeframe this corroborates with the baseline year for Sustainable Development Goals (SDGs). So, the findings reported in this study could be used to track the changes to be reported by subsequent surveys. Post 2015, the government of Nepal has introduced various health financing reforms, notably National Health Insurance (launched in 2016) in an attempt to curb down the financing barriers to healthcare. The scheme, now launched in all 77 districts of Nepal, is fully subsidized for the poor and vulnerable households. So, in the subsequent work we will try to incorporate the findings form the surveys from the years conducted after 2016. This will contribute towards monitoring of the financial risk protection, one of the key dimensions of universal health coverage.

2.	The authors suggest designing risk-pooling arrangements to make progress towards UHC. Will this solve all the problems people face in Nepal, especially the most disadvantaged groups incurring CHE? How about the supply side of the health care system? If there are not enough provisions for these services, how come, UHC will help solve CHE? I would recommend talking about the supply side of the health care system in Nepal.

**Response:** Many thanks for raising this important concern.  We have revised the background section focusing on health financing instruments. Still, we have focused more on the “risk-pooling arrangements” together with “health financing arrangements” since the former is more closely associated with financial protection because  the way revenue for health are generated and pooled affects who bear the burden of healthcare payments and who utilizes the healthcare. So appropriately designed health financing arrangements help make sure people are paying for healthcare based on their ability to pay (socio-economic position in the society) and using the healthcare based on their need (equity). So, health financing arrangements like public health insurance are designed and launched to provide financial protection to vulnerable populations against health shocks (such as CHE).

Here, our aim was to analyze the levels and distribution of financial risk protection, a demand side concept that measures the opportunity cost of  healthcare OOP payments in terms of reduced household consumption of other goods and services.  That is why we did not talk much about the supply side of the health system. The framework of health financing and UHC inherently captures ‘service coverage’ or ‘benefit package’ as key functions or dimensions. The revenue generated and pooled by health financing institutions (example: health insurance agency) is used to purchase healthcare on behalf of the population. The financial power of such institutions can ensure the availability and quality of healthcare.

Since the aim of the paper, as stated earlier, was to talk about one of the dimensions of UHC (financial protection) we were unable to include the aspects of the supply-side of healthcare. However, this should not imply that only the pooling function guarantees UHC. We have reviewed the Manuscript and tried to capture the aspects of supply-side as well, wherever applicable. In addition, we have added this line in the limitation section:

*“Since we aimed to measure financial protection indicators in this paper, detailed discussion of the other dimensions of UHC, such as the supply side of the health system, was outside the scope of  the current work.”*

3.	Introduction: "In the last few decades, many low-and middle-income countries (LMIC) have been reforming their health financing arrangements for UHC (4, 5). Countries such as Thailand, Ghana, Tanzania, and Indonesia have successfully introduced the risk-pooling arrangements to the occupation groups of both the formal and informal sector of economy (4-6)."

a.	Turkey introduced universal health coverage in 2008 and offered a good example of such countries that implemented UHC successfully. Therefore, I would suggest including the below references in the text as well to mention Turkey's experience:

Ti̇rgi̇l, A., & Özbuğday, F. C. (2020). Does Public Health Insurance Provide Financial Protection Against Out-Of-Pocket Health Payments? Evidence from Turkey. Sosyoekonomi, 28(45), 11-24.

Atun, R., Aydın, S., Chakraborty, S., Sümer, S., Aran, M., Gürol, I., ... & Akdağ, R. (2013). Universal health coverage in Turkey: enhancement of equity. The Lancet, 382(9886), 65-99.

**Response:** We appreciate your suggestion to expand our literature and references. In the revised manuscript we have included Turkey's example as well. Following text is added in the ‘Introduction’ section:

“*Turkey introduced a non-contributory health insurance scheme named Green Card in 1992 (further revised in 2005 to include outpatient services and pharmaceuticals) to provide financial protection to poor households that were previously not covered by the formal health insurance scheme. Health system and financing reforms under Health Transformation Program in Turkey are successful in reducing inequity in health outcomes and making progress towards universal coverage*.”

4.	"There are two commonly used approaches to measure the extent of financial hardship and financial protection in healthcare: (i) catastrophic health expenditure (CHE) and (ii)impoverishment due to healthcare out-of-pocket (OOP) payments."

Other studies in the literature look at causal mechanisms in terms of catastrophic health expenditures. Therefore, I suggest that the authors provide references to the relevant literature. For example,

Miller, G., Pinto, D. M., & Vera-Hernández, M. (2009). Risk protection, service use, and health outcomes under Colombia's health insurance program for the poor (No. w15456). National Bureau of Economic Research.

**Response**: We followed the examples from WHO and World Bank who used catastrophic health expenditure (CHE) and impoverishment due to healthcare out-of-pocket (OOP) payments as approaches to measure the extent of financial hardship and financial protection in healthcare (see Global Monitoring Report on Financial Protection in Health series such as this <https://www.who.int/publications/i/item/9789240040953>. We used the approaches described in these monitoring reports to measure financial protection at different thresholds. Adopting their approach allowed for comparison across countries and years since the level/measure of the financial risk may depend on the methodology and definition used.

5.	There is much research concerning CHE using these two approaches authors utilize. I do not see any contribution to the literature regarding the approaches taken. However, the authors state that it is the first study to look at CHE, disaggregated at occupations and regions in South-East Asia, which can be accepted as a contribution.

**Response:** We agree with the reviewer that we did not introduce any new methodological approaches to measure financial protection in health. Rather, we used standard approaches to measure CHE and impoverishment due to OOPs commonly found in World Bank and WHO reports. However, the novelty of our work lies in the fact that we estimated the financial risk protection indicators at different occupation groups and geography (provinces, rural/urban). This will be particularly important given that Nepal has transitioned to Federal structure since 2017 from the previous unitary government structure and the government has also launched social health protection schemes targeted to different occupation groups (Example: Social Security Schemes launched targeting formal private sector, National Health Insurance launched targeting the informal workforce).

**Response to the comments from reviewer 2**

Thank you for raising general issues with the paper, we have now tried to address these points including cohesive story around findings and the grammatical aspect. Below are the response to each specific points.

1. Introduction: Please solidify the rationale for your study. It’s stated that there is no disaggregated information (which is an important rationale) but can substantiate with why the disaggregated information would be useful and how it may contribute to the literature or in policy decision processes. Some of these points are listed as bullet points (in page 5) but can be developed and simplified further for clarity.

**[Response]** We thank the reviewer for important suggestions on potential contributions to the existing literature and relevance to policy makers. We have improved the rationale addressing these points in the following text in the last paragraph of the introduction section of the paper.

*…In this context, our study aims to examine the financial protection statistics, both levels and distribution, across occupation and geography using the nationally representative Annual Household Survey (AHS).  Currently, such measures are available only at the aggregate/national level. Besides this, the results of this study can be useful in several ways. First, the GoN through social protection schemes targets the population in different occupations. Therefore, this study will provide baseline, disaggregated estimates of financial protection to monitor the performance of existing risk-pooling arrangements. Second, the financial protection of occupations engaged in informal sectors (which occupies 62% share) (8) is particularly unknown. The coverage of these groups in existing social health protection schemes is also poor (19). Having evidence on financial protection can be useful to policy makers to devise strategies, garner budgetary requirements and monitor effectiveness of the policies over time. Third, rural-urban and provincial disaggregation is important in light of the fact that the country's federal structure provides provincial, local planners & policymakers a significant budgetary discretion in devising strategies to improve financial protection. Fourth, occupational classification indicators move together with the size and composition of the economy, which is an important precondition for progress towards UHC (4, 29). Fifth, each occupation group covers workers falling on a range in a socio-economic spectrum; therefore, it is relevant to check, within each occupation group, whether the financial protection statistics are concentrated more among the rich or poor. This piece of evidence can be useful in devising strategies to bridge the rich-poor gap in financial protection within each occupation group, an important aim of UHC….*

 Methods: Independent variables for what? It would be clearer to first describe what you intend to do and then describe the independent (and dependent) variables rather than starting by calling out independent variables.

**[Response]** We consider this a reasonable point and hence improved the flow of the methodology section as advised by the reviewer, first by highlighting our main dependent variable – financial protection, its measurement – and, then listed the independent variables that are suitable given the aim of this paper and data availability.

2. • Measures of consumptions- not well defined/explained. Elaborate for clarity. What are the different approaches supposed to mean? Are you testing or validating the results with different approaches? If so, you may want to highlight any differences or deviations in findings between the approaches? Also how do you console your findings if you find differences? In the discussion section there is some elaboration on why the differences were observed and the justification (which is good). You may want to state the intention of using different measures in the beginning to help readers follow through the manuscript. For example, what do the different thresholds mean? Why use multiple thresholds/measures and what they mean?

**[Response]** We thank the reviewer for highlighting the important point. Measurement of financial protection, besides healthcare OOP payments, requires a measure of household resources available-to-pay. In the absence of an ideal definition of household resources available-to-pay, there are commonly used proxy measures for it. For these proxy measures, the starting point is a measure of household consumption expenditure. We have now elaborated the components of household consumption expenditure as follows, and added references that provide details about it for interested readers.

*….The AHS used the Classification of Individual Consumption by Purpose (COICOP) to classify household consumption expenditures into 12 broad categories. The categories cover food (COICOP-1 and 11), and non-food consumption expenditures (COICOP 2, 12) that includes tobacco and alcohol, clothing, housing, furnishing, transportation, communication, entertainment, health, education, and other expenses. The details about the components of consumption are available in the AHS report (41)….*

In the literature of catastrophic health expenditure, there is no ideal approach of measurement. The benefit of using multiple approaches is that one measurement approach captures shortcomings of the other, and with collective interpretation, the author can make a rich analysis of the findings and can be useful to the intended audience. In the methodology section, besides elaborating each measurement approach, we have added the significance of multiple approaches, which will be used in the ‘findings’ and ‘discussion’ sections of the paper. We have added the following text to address this issue.

*…The argument behind deducing a certain amount from the total consumption in (ii) and (iii) definition is to capture a better measure of a household’s ability or capacity-to-pay healthcare OOP payments (39). With the (i) definition, the CHE is usually less concentrated among “poor people” (or more concentrated among “rich people”). In order to address this bias at lower tail of income distribution, a pro-poor measure called the CTP approach is used that records a higher incidence of CHE spending among the poor than the budget share approach. A comparative analysis of these two approaches provides a balanced understanding of CHE (40). We used the first two approaches to measure financial protection indicators so that the results will be comparable to the available literature including the global reports on financial protection published by the WHO and the World Bank (8)….*

As elaborated in the ‘methods’ section, shortcoming of one measurement approach is fulfilled by the other and hence reporting them together provides a complete picture. Similarly, we have also highlighted differences between the measurement approaches and their usefulness in measuring financial protection. Additionally, the reasons for using multiple thresholds is also elaborated in the paper (in methodology section).

3. • Results: The tables are a bit long, but they provide a comprehensive descriptive summary (Tables 3, 4). In table 3, are the estimates of consumption per capita or per household. Please clarify.

**[Response]:** Thank you. We agree with the reviewer, the numbers indicate per capita per year and have changed the title accordingly in the revised version.

4. • The results section is too elaborate and dry. It could be condensed by focusing only on the key findings and referring to the tables for the others. What would be more useful for readers is to understand the context and the interpretation of the findings which to some extent is covered in the discussion section. A cohesive story/interpretation from the descriptive findings is lacking. I would suggest building that which would enhance the utility of these kinds of studies.

**[Response]** A very pertinent remark by the reviewer. The CHE, measured using two approaches, provides a comprehensive understanding of the welfare impact of healthcare OOP payments. Therefore, we have stated the important findings based on both budgetshare and CTP approach. These findings, together with impoverishment impact at different poverty lines, will be useful in the discussion. Based on reviewers’ suggestions, we have made the following changes in the findings section.

1.     For table 2 and 3, we have highlighted the important differences that provide merit on its own, and are useful in explaining the observed differences shown in table 4 and 5, leaving the rest of the findings in the table.

2.     For table 4 and 5, we have now highlighted the main findings/statistics related to financial protection highlighting different measurement approaches together so that readers can understand the meaning considering the facts highlighted in the methodology section. We have again considered these key findings in the discussion section.

5. • Why would you expect differences by sex of household head, education status and all of what you refer as independent variables? Elaborating this would help contextualize the results.

**[Response]** Important point! Our aim is to highlight and explain the observed difference in financial protection across occupation and geography. We also got disaggregation across a set of sociodemographic characteristics – household head’s sex, household head’s education and consumption quintile of household. The choice of these variables is based on the review of the latest Labour Force Survey (LFS) 2018/19, which reported that occupation groups differ across gender, education and socioeconomic variables. So, disaggregating both occupation groups and financial protection across sociodemographic characteristics is helpful to explain if such socio-economic variation in occupation categories also explains the differences in financial protection across occupations. We have used these statistics in the discussion section wherever relevant.

6. • Interesting finding that both CHE and impoverishment impact were higher among the richer population in Bagmati province, compared to other. Why would that be the case?

**[Response]** Yes, the elaboration will be helpful for readers.  These statistics reveal important aspects about how CHE, at different thresholds, and impoverishment impact can be used to explain this observed difference. We have added the following text to explain this observation.

*…Over the provinces, all the values of the concentration index were positive for Bagmati province. This indicates that both CHE and impoverishment impacts were disproportionately concentrated among the richer segment of the population. This occurs because a persistently high CHE even at 25% threshold (Table 4) and a nominal percentage of impoverishment impact (Table 5) together indicate that CHE, when occurs, is mostly concentrated among the richer segment in the Bagmati Province…*

7. • If the access to health care is limited, then the CHE is likely going to be small. But how will this lead to impoverishment? Not clear, additional explanation would be helpful (page 20)

**[Response]** This is a policy relevant observation in the financial protection literature. In general, those households lying slightly above the poverty line are not poor. It is commonly reported that such households have poor access to healthcare as the majority of them face different barriers to access healthcare. We have added the following text in the discussion to explain it.

*…The quintile distribution of elementary occupation reveals that more than half (55%) of the households with elementary occupations lie in the bottom two consumption quintiles (Table 2). This indicates that per capita consumption for the majority of such households is marginally above the poverty line before healthcare OOP payments. And, any healthcare OOP (not more than 10% of the total consumption which does not cause CHE) is likely to lower the consumption per capita net of healthcare OOP less than poverty lines thereby impoverishing them without incurring any CHE. This pattern is commonly observed in the poor segment of the population (47)….*

8. • Discussion: No systematic differences in catastrophic health expend across residence by using one method (budget share approach) but some difference using another (CTP). Why would that be the case? How do you explain the differences?

**[Response]** Thank you for indicating the important point. This finding reveals the usefulness of pro-poor measure of CHE. We have explained this observation by adding the following text in the discussion.

*….Across the area of residence, using the budget share approach, we observed no clear difference in CHE, but using the CTP approach, 5% and 3% of households were catastrophic in the rural and urban areas, respectively. The CTP approach deduces food consumption expenditure from the total consumption when measuring CHE. NLSS-III shows that the food share of total consumption is higher in rural areas (54).  The CTP approach is, therefore, a pro-poor measure(40) and provides a better measure of CHE and hence explains observed higher level CHE in rural areas when budget share approach does not provide any evidence of systematic differences.  In the same line, impoverishing health expenditure was quite high in rural areas across all the definitions of poverty lines. …*

9. • Conclusion: There is too much of information in the results/discussion section, prioritization of some key takeaways would be useful to include in the conclusion section rather than restating some of the result figures.

**[Response]:** Now we have reframed the conclusion to explain the key takeaway from the paper.

*….Despite the global and national efforts to reduce the financial protection through various health financing reforms, we observed that financial protection is unequal among and within occupation groups and geography in Nepal. The formal occupations – having lower share of total occupations in Nepal – are protected to a greater degree from the financial hardship resulting from healthcare OOP payments than the informal occupations such as agricultural workers and ‘plant operators and craft workers’ that occupy two thirds of all the occupations. Additionally, to make things worse, the problem is further concentrated in poorer segments of informal occupations. We also observed a high CHE in relatively richer provinces and rural areas when adjusted with pro-poor measurement approaches. On the other hand, the impoverishment impact was found high in relatively poorer provinces and rural areas. These findings suggest a need for policies and strategies that generate incentives for effective uptake of the informal occupations in the risk-pooling arrangements, and also address the provincial and urban/rural differences in financial protection by harnessing the benefits of federal structural and independent local units….*
