# Supplementary material for: Occupational and geographical differentials in financial protection against healthcare out-of-pocket payments in Nepal: Evidence for universal health coverage
Source: PLoS One. 2023 Jan 27;18(1):e0280840. doi: 10.1371/journal.pone.0280840 (PMC9882914; doi:10.1371/journal.pone.0280840)
Supplement: Attachment — Submitted filename: Comments.docx [file pone.0280840.s004.docx]

The paper is a good implementation of analysis of financial hardship. The authors did a good analysis of catastrophic expenditure, even comparing budget share approach and CT. The methods used are in line with WHO and World Bank publications on this topic. The results are also in line with what is expected of these types of analysis.

The paper could improve if discussions could be deepened. Right now, it basically says households belonging to informal occupations were more prone to catastrophic and impoverishing expenditures. But that finding is well established already in the literature, especially so that informality is very much highly correlated with education level and income levels. The real question is what should be done about it? Given that this is a country level paper, it could have delved deeper into answering this question, but the paper brushed it aside to the limitations of the study, which is quite unfortunate. For this to be worthy of publication in Plos one, strengthen the abstract and paper to highlight what is new? The disaggregation by occupation is new but then go an extra step and link it to how it can be useful for policy making in Nepal. Cite some initiatives that could be done in Nepal to apply this finding. In the response to the reviewer comments, the authors were mentioning that Nepal has transitioned to Federal Structure since 2017 and has launched social protection schemes—discuss these in the paper. How does this link to your findings?

(1) The first paragraph in the introduction seems misplaced. It talks about Thailand, Turkey, etc. when the paper is about Nepal. It will better to talk about Nepal first to put it in context, before talking about other countries.
(2) In the discussion of results, discuss about foregone care. One of the reasons why there might be low catastrophic and impoverishing expenditures among the poor is because they did not seek care, even when they needed to. This is one of the weaknesses of these indicators. If impossible to capture foregone care or unmet need using the survey data, the paper should at least acknowledge that this could exist.
(3) Another indicator that was not mentioned in the paper are those who are already poor even before health expenditure, and when they spent for health, they became pushed into poverty even further. As the 2021 global monitoring report of WHO and WB showed, this number is even higher than those who were impoverished due to healthcare. The paper should examine this indicator since they are able to do this with the data they have.

Minor points:

(1) In table 1, add a note to say what is the value in parenthesis.
(2) Typo page 14. “Similar pattern was observed among salaries…” should it be salaried?
(3) Type page 14. There are two periods before “In Gandaki…”
