# Supplementary material for: Occupational and geographical differentials in financial protection against healthcare out-of-pocket payments in Nepal: Evidence for universal health coverage
Source: PLoS One. 2023 Jan 27;18(1):e0280840. doi: 10.1371/journal.pone.0280840 (PMC9882914; doi:10.1371/journal.pone.0280840)
Supplement: Attachment — Submitted filename: Response to Reviewers_R2.docx [file pone.0280840.s005.docx]

Date: 06/01/2023

**Occupational and geographical differentials in financial protection against healthcare out-of-pocket payments in Nepal: evidence for universal health coverage**

**PONE-D-22-17102R1**

To,

The Academic Editor

PLOS One

**Subject: Rebuttal letter to the manuscript PONE-D-22-17102 in second round of revision.**

Dear Sir

Thank you very much for a thorough review by the academic editor and reviewers – the points raised provided us an opportunity to improve the manuscript. Below, we have provided a point-by-point response to the issues raised by the academic editor and reviewers. The comments are in normal font, our response is in blue font and in-text additions to address the comments are in green fonts. All the in-text additions are available in the revised manuscripts in track change.

**Editor’s comment**

Thank you for submitting your revised manuscript to PLoS ONE. I have carefully read your revised manuscript and responses to the previous round of review comments. I appreciate your efforts. Nonetheless, one reviewer had raised several vital concerns regarding your study, and had provided suggestions to improve your work further, particularly the Discussion section. Basically, I am in agreement with the reviewer’s concerns. In light of the concerns, I invite you to respond to the reviewer's comments and resubmit a revised manuscript. Please respond carefully and thoroughly to each comment of the reviewer. Please explain where you feel you cannot completely agree with the reviewer’s suggestions.

**Response:** Dear Editor, we are grateful to you and the third reviewer for providing us an opportunity to revise our manuscript. We are also thankful to two anonymous reviewers of the previous round for providing valuable comments. We have tried our best to further revise our manuscript based on the additional comments provided by the third reviewer. A point-by-point response to all the comments are available below. The intext additions in the manuscript are available in green font.

**Reviewer’s comment**

The paper is a good implementation of analysis of financial hardship. The authors did a good analysis of catastrophic expenditure, even comparing budget share approach and CT. The methods used are in line with WHO and World Bank publications on this topic. The results are also in line with what is expected of these types of analysis.

**Response:** Many thanks for this complement.

The paper could improve if discussions could be deepened. Right now, it basically says households belonging to informal occupations were more prone to catastrophic and impoverishing expenditures. But that finding is well established already in the literature, especially so that informality is very much highly correlated with education level and income levels. The real question is what should be done about it? Given that this is a country level paper, it could have delved deeper into answering this question, but the paper brushed it aside to the limitations of the study, which is quite unfortunate.

**Response:** Dear Reviewer, many thanks for this observation. As per your advice, we have further elaborated our discussion to include the possible strategies that policy makers can adopt to address the observation that the households belonging to informal occupations were more prone to catastrophic and impoverishing healthcare expenditures. The following sentences are added in the fourth paragraph of the discussion section.

“As a response to this situation, the government of Nepal has launched many social protection schemes focusing particularly towards poor and vulnerable population groups belonging to the informal sector. A few examples are: the implementation of national health insurance, impoverished citizen treatment scheme, and free basic health service package. National health insurance is fully subsidised for identified poor households. However, the result of the poor household identification survey is available for only 26 districts. This has put poor households from remaining districts in a disadvantaged position as they are not being able to benefit from this policy decision. For those households who are enrolled (either through contribution or subsidy) due to inequitable distribution of healthcare providers across provinces, people have to travel to a nearby city or Kathmandu where the cost of treatment could be higher than that could have been in the nearby area. Public facilities in remote areas are understaffed and frequently face a stock out of medicines. Due to this, the people in such areas either have to rely on private clinics and tertiary hospitals in the cities. Due to all these reasons, people from remote and rural areas, who are also from the informal sector, get disproportionately disadvantaged. To tackle this poor households from all the districts should be identified so that they can benefit from public subsidies, healthcare should be more equitably distributed and human resources and logistics should be ensured in the rural and remote areas. The three tiers of government should work together so that the households eligible for health insurance subsidies could be identified and basic health services should be insured at the local level so that people do not have to pay a large sum of money to private and tertiary hospitals even to cure minor ailments.”

For this to be worthy of publication in Plos one, strengthen the abstract and paper to highlight what is new? The disaggregation by occupation is new but then go an extra step and link it to how it can be useful for policy making in Nepal. Cite some initiatives that could be done in Nepal to apply this finding.

**Response:**

Man thanks for this feedback. We have made the following changes in the manuscript to accommodate your input.

a. We have rephrased the Methods section of the ‘Abstract’ as below in the revised manuscript:

“We measured catastrophic health expenditure (CHE) due to OOP using budget share and capacity-to-pay approach, impoverishment impact at absolute and relative poverty lines. This study is the first of its kind from south-east Asia to analyse disaggregated estimates of financial protection across occupations and geography.”

b. We have added the following sentences in the ‘Materials and method’ section:

“ This study is the first of its kind from south-east Asia to analyse disaggregated estimates of financial protection across occupations and geography using standard methods.”

In addition, we have already highlighted the points mentioned in ‘a’ and ‘b’ in the Discussion section in the original submission as well as in the fourth paragraph of the ‘Introduction’ section.

c. The last paragraph of the ‘Introduction’ section in the original submission provides some background on how this analysis can be useful at policy level. In addition, we have added the following paragraph in the ‘Discussion’ (1st paragraph after the first line) section of revised manuscript to show how disaggregation of financial protection results across occupations could be useful for policymaking in Nepal:

“The disaggregation of results across occupations could be helpful for policymakers in Nepal in the following ways. First, it shows different levels of vulnerability faced by different occupation groups, and thereby identifies the most vulnerable groups that need government intervention. Second, it provides figures to monitor the financial protection of different occupational groups and assess whether public policies are reducing the inequitable distribution of social security measures. Third, it provides a rationale for nationwide risk pooling across occupation groups since the current pooling arrangements through National Health Insurance and Social Security Fund are more focused in the informal sector and formal private sector, respectively.”

In the response to the reviewer comments, the authors were mentioning that Nepal has transitioned to Federal Structure since 2017 and has launched social protection schemes—discuss these in the paper. How does this link to your findings?

**Response:** We thank the reviewer for this suggestion. We have updated the sixth paragraph of the ‘Discussion’ section by adding the following details:

“The federal structure of the country provides opportunity to provincial and local governments to develop community-based targeting of poor households in the absence of poor-households identification surveys from the federal government. This will help poor households from the rest of the districts benefit from the health insurance subsidy. To improve cross-subsidization health insurance schemes can be made mandatory for both formal and informal sectors of workers. Local governments are in a good position to enforce mandatory enrolment by linking the membership with other social services and benefits provided through the local units (wards and municipalities).”

**Specific comments**

(1) The first paragraph in the introduction seems misplaced. It talks about Thailand, Turkey, etc. when the paper is about Nepal. It will better to talk about Nepal first to put it in context, before talking about other countries.

**Response:** Many thanks for this suggestion. Our aim is to analyse financial protection together with standard occupational classification. In order to set a general rationale for such analysis in the global context, we started with the cases of those countries that are making good progress in UHC, and they have tied the healthcare financing arrangements to the different occupations relevant to their economy. Before talking about Nepal, it was necessary to provide an overview of how other countries are reforming their health system and health financing arrangement to minimise the financial burden of their citizens while using healthcare. In Nepal, healthcare financing arrangements appear to cover different occupation groups as well - a further specific case from the global observation. Therefore, in the first paragraph, we started with an overview of financial protection for UHC and its relation with occupational groups in a global context. Then, we proceeded with the case of Nepal including potential relevance to the policy makers.

(2) In the discussion of results, discuss about foregone care. One of the reasons why there might be low catastrophic and impoverishing expenditures among the poor is because they did not seek care, even when they needed to. This is one of the weaknesses of these indicators. If impossible to capture foregone care or unmet need using the survey data, the paper should at least acknowledge that this could exist.

**Response:** Many thanks for this important concern. We have made following changes in the revised manuscript so that the foregone care is acknowledged.

a. We have modified fourth paragraph of Discussion section as follow:

“These circumstances made healthcare less affordable to them, *so the workers belonging to informal sector may have forgone care due to financial barriers*. This is one of the reasons for the relatively lower level of CHE in this sub-group.

b. We have added following sentence in the ‘Limitation of this study’

Fourth, because of the data unavailability in the survey, we could not provide an account of foregone care – an important and complementary analysis to the financial protection measures.

(3) Another indicator that was not mentioned in the paper are those who are already poor even before health expenditure, and when they spent for health, they became pushed into poverty even further. As the 2021 global monitoring report of WHO and WB showed, this number is even higher than those who were impoverished due to healthcare. The paper should examine this indicator since they are able to do this with the data they have.

**Response:** We acknowledge this suggestion. We have included a new table (Table 6) to show the proportion of households pushed further down the poverty across background characteristics and using both the absolute and relative poverty lines. We have added the following paragraph in the ‘Results’ section.

“Table 6 shows the share of population (those already below the poverty line) further pushed into poverty due to healthcare OOP expenditure. At the national level, an estimated 0.38% of the population was pushed further below the extreme poverty line of purchasing-power-parity US $1.90 per capita per day. At US $ 3.2 per capita per day, an estimated 1.25% of the population was pushed further down the poverty line due to healthcare OOP expenditure. Using relative poverty lines of 50% and 60% of median consumption estimated 0.39% and 0.68% of the population, respectively, was further pushed down the poverty due to healthcare OOP. Among household occupation groups, the percentage of the population pushed further down the poverty due to healthcare OOP was least for ‘managers and professionals’ and ‘clerical and support worker’, whereas the percentage was highest for ‘Agriculture’ and ‘Elementary occupations’ at both absolute and relative poverty lines.

Disproportionately greater share of the population from rural areas were pushed further down the poverty compared to the rural area across all the definitions of poverty. Similarly, a larger share of the population from Madhesh, Karnali and Sudurpaschim provinces were disproportionately pushed further down the poverty line compared to other provinces. There was a slight difference in terms of sex of household head, more male headed households were further pushed down the poverty compared to female headed households.

Across the four education categories, the proportion of households pushed further down the poverty was least when the head of household had higher education. Proportion of households pushed further down the poverty increased gradually from least to highest when the household head’s education changed from higher education to less than primary education. Similarly, the share of households pushed further down the poverty increased monotonically from highest wealth quintile to lowest wealth quintile. These observations were consistent with all the definitions of poverty.”

In addition, we have also updated the ‘Discussion’ and ‘Conclusion’ to incorporate the findings shown in Table 6.

**Minor points:**

(1) In table 1, add a note to say what is the value in parenthesis.

**Response:** Many thanks for the observation. We have provided a note at the bottom of table 1 and 2.

(2) Typo page 14. “Similar pattern was observed among salaries…” should it be salaried?

**Response:** Many thanks for the observation. Corrected in the revised manuscript.

(3) Type page 14. There are two periods before “In Gandaki…”

**Response:** Many thanks for the observation. Corrected in the revised manuscript.

We believe we were able to satisfactorily address all the comments from reviewers and made necessary corrections in the manuscript.

Kind regards,

Authors.
